# First person – Emily Harders

**DOI:** 10.1242/bio.060303

**Published:** 2024-01-25

## Abstract

First Person is a series of interviews with the first authors of a selection of papers published in Biology Open, helping researchers promote themselves alongside their papers. Emily Harders is first author on ‘ Avian extraembryonic membranes respond to yolk corticosterone early in development’, published in BiO. Emily conducted the research described in this article while a master's student in Dr Ryan Paitz's lab at Illinois State University, Normal, USA. She is now currently a specimen processing technician at Nebraska Medicine in Omaha, Nebraska, while she applies for PhD programs. She is interested in understanding how maternal/fetal signalling is regulated in extraembryonic tissues.


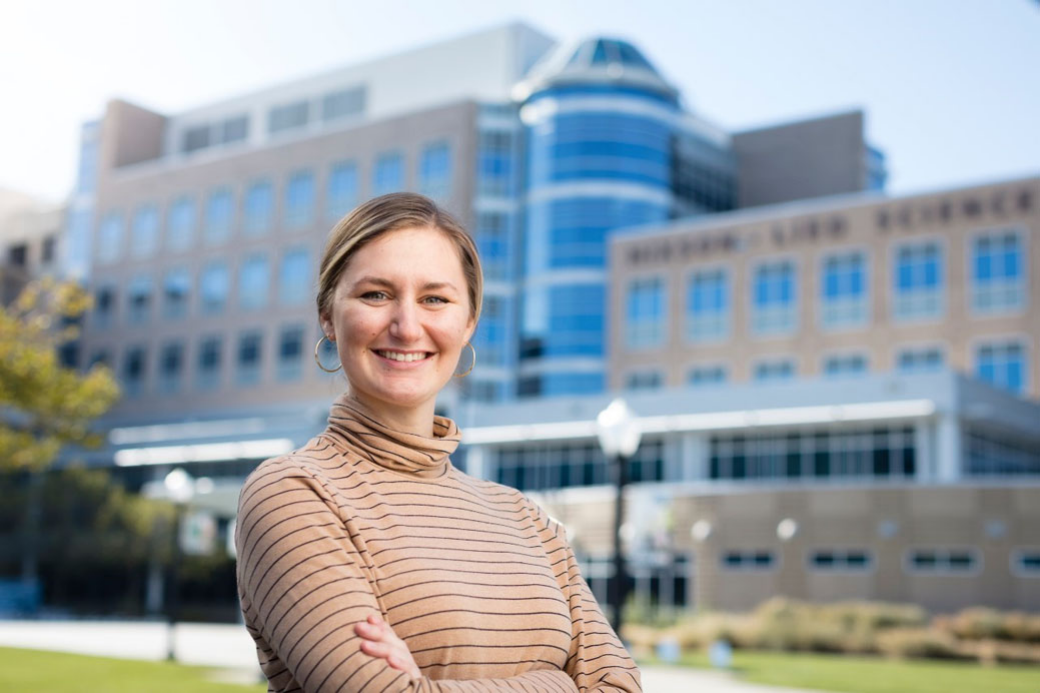


**Emily Harders**

**Describe your scientific journey and your current research focus**

My scientific journey so far has progressively built upon itself. I began by exploring the impact of long-term parasitism by the horsehair worm on host cricket physiology during my bachelor's degree. I became fascinated with the physiological processes involved when two organisms are in physical contact for long periods of time and began drawing parallels to the manipulative dynamics at play in maternal-fetal interactions. During my master's I explored this and studied maternal-fetal interactions in the context of maternal stress. I focused on how the extraembryonic membranes in chicken eggs metabolize and respond to glucocorticoids, which are deposited in the yolk during times of maternal stress. While looking towards a PhD, I am fascinated by the idea that extraembryonic membranes are the physical link between generations and their function can be affected by maternal environment and physiology, permanently altering the development and lifelong health of the next generation.

**Who or what inspired you to become a scientist?**

As a first-generation college student from a rural community, the only scientific role models I had before my bachelor's degree were healthcare professionals. However, once at university, I was exposed to a whole new scientific world and experienced the scientific process myself through three years of undergraduate research. During my master's degree, I further honed in on my research interests, and the independence and curiosity I have while conducting research resonates with me and aligns with the entrepreneurial spirit instilled in me by my parents, both business owners.

**Which part of this research project was the most rewarding?**

The most rewarding part of my research has been presenting my findings at conferences. I enjoy telling a story through my research and then discussing my findings with other scientists who offer new perspectives. My oral presentation at the 2023 Society of Integrative and Comparative Biology's national meeting was very memorable as it was my first oral presentation at a large national conference. I was awarded the Aubrey Gorbman award for best student oral presentation in the Division of Comparative Endocrinology, and being recognized by current endocrinologists made me feel very proud of my work, welcomed by a community of scientists, and confident that I could have a tangible, successful career in this field.

**Figure:**
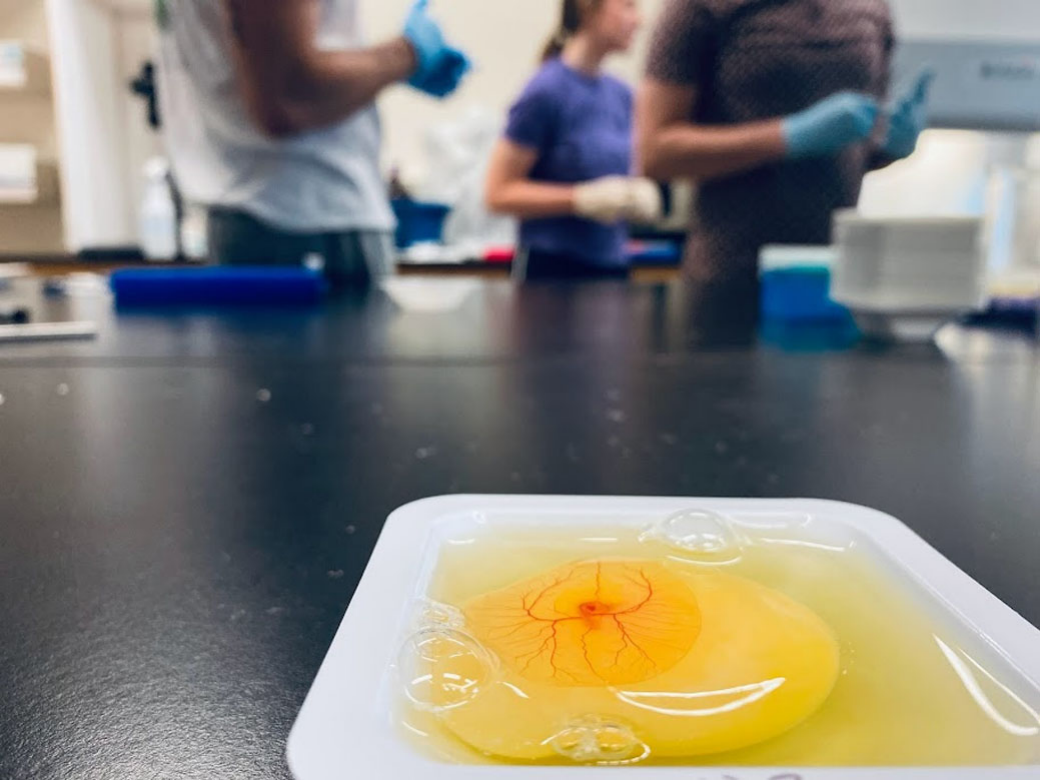
An ‘egg-cellent’ day in the lab.

**What do you enjoy most about being an early-career researcher?**

As an early-career researcher, I have enjoyed the diversity of research areas I have been able to delve into. I started off studying ecology, evolution, and behaviour using crickets in my bachelor's, then transitioned to studying molecular biology in mice as a lab technician. For my master's, I studied developmental endocrinology using chicken eggs as a model. Having this wide range of experiences has expanded my perspective and made me a more well-rounded scientist. Not only that but at this early-career stage, my experiences are a foundation for me to reflect on and discern what I am most passionate about as I apply for PhD programs.

**What piece of advice would you give to the next generation of researchers?**

My advice to the next generation of researchers would be to advocate for yourself and not hesitate in expressing your goals. I've found that many opportunities have presented themselves simply by asking.

I've found that many opportunities have presented themselves simply by asking.

**What's next for you?**

I am planning on starting a PhD program fall of 2024 studying maternal/fetal communication within the placenta.
